# Auditory localization should be considered as a sign of minimally conscious state based on multimodal findings

**DOI:** 10.1093/braincomms/fcaa195

**Published:** 2020-12-12

**Authors:** Manon Carrière, Helena Cassol, Charlène Aubinet, Rajanikant Panda, Aurore Thibaut, Stephen K Larroque, Jessica Simon, Charlotte Martial, Mohamed A Bahri, Camille Chatelle, Géraldine Martens, Srivas Chennu, Steven Laureys, Olivia Gosseries

**Affiliations:** 1 Coma Science Group, GIGA-Consciousness, University of Liège, 4000 Liège, Belgium; 2 Centre du Cerveau^2^, University Hospital of Liège, 4000 Liège, Belgium; 3 Psychology and Neurosciences of Cognition PsyNCogn, University of Liège, 4000 Liège, Belgium; 4 GIGA-Cyclotron Research Centre-In Vivo Imaging, University of Liège, 4000 Liège, Belgium; 5 School of Computing, University of Kent, Chatam Maritime ME4 4AG, UK; 6 Department of Clinical Neurosciences, University of Cambridge, Cambridge CB2 OQQ, UK

**Keywords:** disorders of consciousness, auditory localization, diagnosis, brain imaging, electroencephalography

## Abstract

Auditory localization (i.e. turning the head and/or the eyes towards an auditory stimulus) is often part of the clinical evaluation of patients recovering from coma. The objective of this study is to determine whether auditory localization could be considered as a new sign of minimally conscious state, using a multimodal approach. The presence of auditory localization and the clinical outcome at 2 years of follow-up were evaluated in 186 patients with severe brain injury, including 64 with unresponsive wakefulness syndrome, 28 in minimally conscious state minus, 71 in minimally conscious state plus and 23 who emerged from the minimally conscious state. Brain metabolism, functional connectivity and graph theory measures were investigated by means of ^18^F-fluorodeoxyglucose positron emission tomography, functional MRI and high-density electroencephalography in two subgroups of unresponsive patients, with and without auditory localization. These two subgroups were also compared to a subgroup of patients in minimally conscious state minus. Auditory localization was observed in 13% of unresponsive patients, 46% of patients in minimally conscious state minus, 62% of patients in minimally conscious state plus and 78% of patients who emerged from the minimally conscious state. The probability to observe an auditory localization increased along with the level of consciousness, and the presence of auditory localization could predict the level of consciousness. Patients with auditory localization had higher survival rates (at 2-year follow-up) than those without localization. Differences in brain function were found between unresponsive patients with and without auditory localization. Higher connectivity in unresponsive patients with auditory localization was measured between the fronto-parietal network and secondary visual areas, and in the alpha band electroencephalography network. Moreover, patients in minimally conscious state minus significantly differed from unresponsive patients without auditory localization in terms of brain metabolism and alpha network centrality, whereas no difference was found with unresponsive patients who presented auditory localization. Our multimodal findings suggest differences in brain function between unresponsive patients with and without auditory localization, which support our hypothesis that auditory localization should be considered as a new sign of minimally conscious state. Unresponsive patients showing auditory localization should therefore no longer be considered unresponsive but minimally conscious. This would have crucial consequences on these patients’ lives as it would directly impact the therapeutic orientation or end-of-life decisions usually taken based on the diagnosis.

## Teaser

This study provides evidence that auditory localization should be considered as a new sign of minimally conscious state. The prevalence of auditory localization increases with the level of consciousness, and patients with unresponsive wakefulness syndrome and auditory localization have higher brain functioning and better outcome than unresponsive patients who do not show auditory localization.

## Graphical Abstract

**Graphical Abstract:**
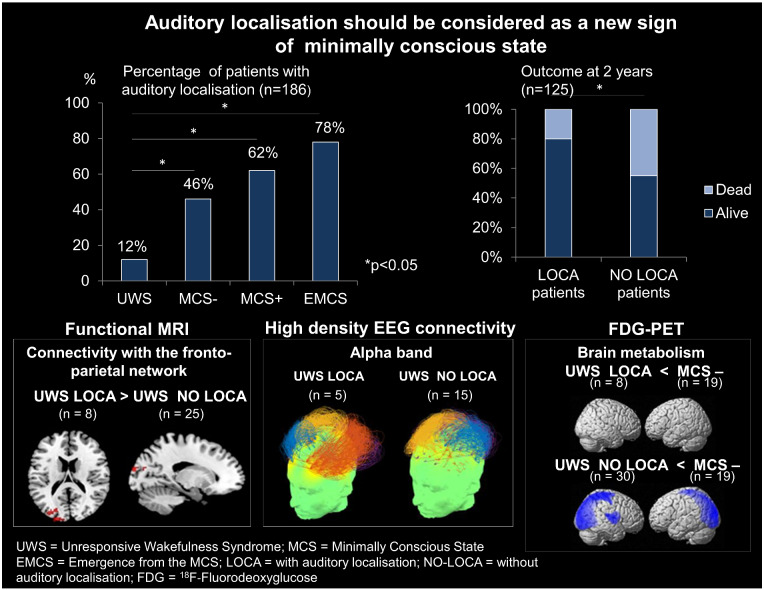

## Introduction

After a period of coma, patients with severe brain injuries may either die or evolve through different states of impaired awareness, referred to as disorders of consciousness (DOC). In the unresponsive wakefulness syndrome (UWS), patients open their eyes but only show reflexive movements (Jennett and Plum, 1972; Laureys *et al.*, 2010). In the minimally conscious state (MCS), patients show reproducible but fluctuating signs of consciousness (Giacino *et al.*, 2002). Within this clinical entity, patients in MCS minus (MCS−) show non-language-related oriented behaviours, such as visual pursuit and pain localization, whereas patients in MCS plus (MCS+) are able to follow simple commands, intelligibly verbalize and/or communicate intentionally (Bruno *et al.*, 2011). Once patients functionally communicate or use objects, they emerge from the MCS (EMCS, Giacino *et al.*, 2002).

An accurate diagnosis of the level of consciousness is crucial, given the implications for prognosis, treatment (e.g. analgesic therapy) and end-of-life decisions (Boly *et al.*, 2008; Demertzi *et al.*, 2011; Faugeras *et al.*, 2018; Thibaut *et al.*, 2019). To this end, clinicians use neurobehavioural scales, and preferably the Coma Recovery Scale-Revised (CRS-R). The CRS-R is a standardized neurobehavioural assessment composed of six subscales (auditory, visual, motor, oromotor/verbal, communication and arousal) which allows the differentiation between patients in MCS and in UWS (Giacino *et al.*, 2004; Seel *et al.*, 2010). Establishing a correct diagnosis may, however, be challenging, since the absence of behavioural responses at the bedside can be due to other factors than the absence of consciousness, such as motor or language deficits (Schnakers *et al.*, 2009; Gosseries *et al.*, 2014).

Although auditory localization is frequently assessed in clinical practice, there is no clear consensus whether it is a purely reflexive or conscious behaviour. According to the Multi Society Task Force on persistent vegetative state in the USA, ‘a turning of the head and eyes towards a peripheral sound’ is considered as an inconsistent primitive auditory orienting reflex (The Multi-Society Task Force on PVS, 1994). The workgroup of the US Aspen Neurobehavioural Conference proposed that an auditory startle and/or a brief orientation to sound correspond to the UWS, whereas ‘localizing a sound location’ should be part of the clinical criteria defining the MCS (Giacino *et al.*, 2002). The guidelines in the UK considered ‘turning fleetingly the eyes towards a loud sound’ a compatible but atypical behaviour of the UWS (Working Party of the Royal College of Physicians, 2003).

Auditory localization is also associated with different clinical entities depending on post-coma scales. In the CRS-R, auditory localization is compatible with the diagnosis of UWS, whereas localization in other sensory modalities (i.e. visual fixation, visual pursuit and localization to noxious stimulation) is considered as a sign of MCS. In the Sensory Modality Assessment Rehabilitation Technique, auditory localization is associated with the diagnosis of either UWS or MCS, based on the quality and the consistency of the answers (Gill-Thwaites and Munday, 2004). In this hierarchical scale, a distinction is made between reflex and localization behaviours, with five levels that range from ‘no response’ (level 1) to ‘reflexive’ (level 2), ‘withdrawal’ (level 3), ‘localizing’ (level 4) and ‘discriminating’ responses (level 5). Finally, the Wessex Head Injury Matrix classifies auditory localization as a social and community interaction, which is considered to be hierarchically superior to reflexive behaviours (Shiel *et al.*, 2000).

One might argue that auditory spatial processing (including auditory localization) should be considered as a reflex because it is known to take place within the brainstem, more particularly in the superior olivary complex (Baehr, 2005). However, recent neuropsychological, neuroimaging and brain stimulation studies support the involvement of the cerebral cortex in this ‘auditory consciousness’, especially the fronto-parietal and fronto-temporal networks (Maeder *et al.*, 2001; Clarke *et al.*, 2002, Lewald *et al.*, 2004*b*). Lesion studies also suggest a cortical involvement in auditory localization (Clarke *et al.*, 2000, 2002). Numerous activation studies using sound localization tasks notably confirmed an important contribution of the temporal, parietal and prefrontal cortices in auditory spatial processing (Bushara *et al.*, 1999; Weeks *et al.*, 1999; Maeder *et al.*, 2001; Brunetti *et al.*, 2005; Brancucci *et al.*, 2016). In sum, all these previous studies suggest that auditory localization requires the contribution of several cortical regions.

Moreover, as pointed out by Naccache (2018), the CRS-R enables to differentiate cortically mediated behaviours from those that are not, with an almost perfect correspondence between MCS/UWS items and cortical/subcortical origin of these behaviours. Indeed, 11 MCS items would reflect cortical activity, whereas 10 UWS items would reflect subcortical activity. As an example, responses to noxious stimulations are of three types: stereotypical, flexion withdrawal and localization (Schnakers and Zasler, 2007; Schnakers *et al.*, 2010). In the CRS-R, the first two correspond to UWS items and are related to brainstem and subcortical processing, whereas localization is known to require cortical activity and hence considered as an MCS item (Porro *et al.*, 2007; Boly *et al.*, 2008). Based on this observation, Naccache (2018) proposed to redefine the MCS as a cortically mediated state that would identify behaviours recruiting cortical networks rather than conscious behaviours *per se*.

This study aims to test the hypothesis that auditory localization is a sign of MCS, and reflects a higher-level cognitive processing in a large sample of patients recovering from coma, using a multimodal approach with clinical, neuroimaging and neurophysiological data. We expect that the probability to observe auditory localization increases with the level of consciousness (as it is the case with visual pursuit, which requires from the patient to visually localize an object/a person) (Giacino and Kalmar, 1997; Dolce *et al.*, 2011). Moreover, we expect that UWS patients showing auditory localization (UWS LOCA) recover better than UWS patients without (UWS NO-LOCA), as previously observed with visual pursuit (Giacino and Kalmar, 1997). We also hypothesize that compared to UWS NO-LOCA patients, UWS LOCA patients have greater brain metabolism (as shown with visual fixation) (Bruno *et al.*, 2010), and higher connectivity in brain areas linked to auditory processing and awareness. Finally, we expect that UWS LOCA patients have a more similar brain activity to MCS− patients, which is the clinical entity that UWS LOCA patients would belong to if auditory localization was considered a new sign of MCS.

## Materials and methods

### Study design and participants

**Figure 1:**
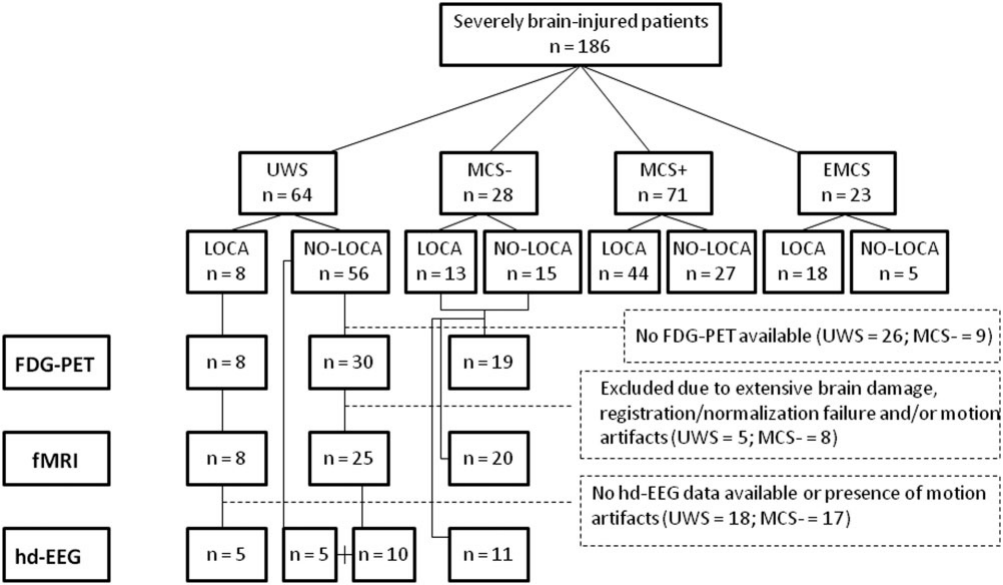
**Flowchart.** One hundred and eighty-six severely brain-injured patients met our inclusion criteria (Supplementary Material I). Among them, 64 were diagnosed in UWS, 28 in MCS−, 71 in MCS+ and 23 in EMCS. The number of patients with and without auditory localization is reported for each clinical entity.

We first investigated the presence of auditory localization and the repeatability of this behaviour in a large sample of 186 severely brain-injured patients (66 females, mean age, 39 ± 16 years) in a prolonged DOC (>28 days post-injury). The behavioural diagnosis of these patients was based on repeated CRS-R assessments (for inclusion criteria, see Supplementary Material I). The median interval between brain injury and assessment was 9 months (range, 1 month–29 years). Etiologies were traumatic brain injury (TBI) in 100 (53.8%) and non-TBI in 86 patients (46.2%). Of these 186 patients, 64 were diagnosed in UWS (34.4%), 28 in MCS− (15%), 71 in MCS+ (38.2%) and 23 in EMCS (12.4%). Next, we considered only the patients in UWS who underwent at least one neuroimaging or electrophysiology-based examination to evaluate the effect of auditory localization on brain activity in patients considered unconscious based on the current gold standard (i.e. the CRS-R) (Giacino *et al.*, 2004). These UWS patients were divided in two groups: LOCA (presence of auditory localization) and NO-LOCA patients (absence of auditory localization). Finally, we compared these two subgroups of UWS patients with a subgroup of MCS− patients. Some of the patients’ data were excluded at the time of the analyses because of severe artefacts or extensive brain damages (i.e. more than two-thirds of one hemisphere) (Fig. 1). Individual demographic and clinical data of UWS and MCS− patients are available in Supplementary Material II. Data on healthy control subjects (HCS) were also collected for each paraclinical examination [i.e. functional MRI (fMRI), FDG-PET and high-density electroencephalography (hd-EEG)]. The study was approved by the institutional ethics committee, and written informed consents were obtained from the patients’ legal representatives and healthy participants.

### Procedure and statistical analyses

#### Behavioural and outcome data

Experienced clinicians conducted at least five assessments with the CRS-R to ensure a reliable diagnosis for each patient (Wannez *et al.*, 2017). Auditory localization was assessed according to the CRS-R guidelines (Supplementary Material IV). Patients were assigned to the group ‘with auditory localization’ if the CRS-R auditory localization item was observed in at least one assessment (out of a minimum of five assessments). The repeatability of auditory localization was defined as the number of CRS-R assessments in which auditory localization was observed, divided by the total number of assessments. Patients were also followed up to 2 years after the assessments using the extended version of the Glasgow Outcome Scale-Extended (GOSE) (Wilson *et al.*, 1998). This scale defines possible functional outcomes after a brain injury, ranging from death, to vegetative state, severe or moderate disability and good recovery. Based on this scale, a score from 1 (death) to 8 (good recovery) was assigned to each patient.

We compared our subgroups of UWS LOCA and UWS NO-LOCA patients with the group of HCS regarding the age and gender using one-way ANOVA and Chi-square tests. We also compared the subgroup of MCS− patients, respectively, with UWS LOCA and NO LOCA patients regarding the aetiology and time since injury using the Fisher’s exact test and Wilcoxon Mann–Whitney test, and regarding the age and gender using independent-sample *t*-test and Fisher’s exact test. Statistical differences for the presence of auditory localization and its repeatability between patient groups were, respectively, examined using Chi-square and Kruskal–Wallis H tests. A multinomial logistic regression was used to predict the level of consciousness (categorical variable with four categories: UWS, MCS−, MCS+ and EMCS) using the age, aetiology, time since injury and auditory localization as explanatory variables. Statistical differences for the clinical outcome (survival and improvement rates) between patients with and without auditory localization were assessed using Fisher’s exact tests. Survival was defined by a score at the GOSE different from 1, whereas improvement was defined by a score at the GOSE > 2 for UWS, >3 for MCS and >4 for EMCS.

#### [^18^F]-fluorodeoxyglucose positron emission tomography

Cerebral metabolic rates for glucose were studied by means of resting [^18^F]-fluorodeoxyglucose positron emission tomography (^18^FDG-PET—Gemini Big Bore TF, Philips Medical Systems) as described elsewhere and in Supplementary Material V (Stender *et al.*, 2014). Data of UWS LOCA (*n* = 8) and NO-LOCA (*n* = 30) patients were compared independently to 34 age-matched HCS (mean age, 43 ± 15 years, 15 women).

Statistical analyses with Statistical Parametric Mapping 12 (SPM12) were used to identify the brain areas of decreased metabolism in LOCA and NO-LOCA patients in UWS compared to HCS, and in LOCA and NO-LOCA patients in UWS compared to patients in MCS−. Both age and time since injury were added as covariates because of group differences. The age covariate was standardized (Kreyszig, 1979; Aho, 2013) before fitting the SPM’s General Linear Model, with a centering to a mean value of 0 by substracting the mean and scaled to a standard deviation of 1 effectively transforming it to a standard score, to allow for the interpretation of potential interaction (Afshartous and Preston, 2011). This nuisance covariate was used for all analyses. Moreover, for analyses directly comparing patient groups, time since injury was taken as a second regressed covariate. This covariate exhibited a skewed distribution on different magnitude orders, which is not optimal for the General Linear Model assumptions of normality of the residuals. Indeed, it is now well accepted that such outliers can inflate the effect sizes of non-robust methods that are relying on the mean and variance (Wilcox and Rousselet, 2018). To overcome this issue, we applied a non-linear data transform consisting of a logarithmic transform (log10), resulting in a ‘log time since injury’ covariate (Bland and Altman, 1996). The results were considered statistically significant at voxel-wise of *P *< 0.05 false-discovery rate (FDR) corrected (whole-brain level) (Friston *et al.*, 1996).

#### Functional MRI

We also acquired structural (T1-weighted 3D gradient echo images) and functional (300 T2*-weighted resting-state volumes) MRI data on a 3T scanner (Siemens Trio Tim), and data were preprocessed using SPM12 (Supplementary Material V).

Statistically, a seed-based approach was performed using the CONN connectivity toolbox version 16b (Whitfield-Gabrieli and Nieto-Castanon, 2012). The seed-based correlation analysis at first-level-extracted fMRI blood–oxygen-level-dependent time series from a region or a set of regions of interest and determined the temporal correlation between this signal and the time series from all other brain voxels. This process was repeated for each subject and each region of interest. More specifically, we investigated networks, which were defined as the average effect (i.e. uniformly weighted contrast) from a set of regions of interest (which is equivalent to having one region of interest covering the entire network): the auditory network, the default-mode network (DMN) and the fronto-parietal network (FPN), in LOCA and NO-LOCA patients in UWS, in MCS− patients as well as in a group of 36 HCS (mean age, 45 ± 16 years, 13 women). These networks were chosen for their involvement in audition, internal and external awareness, respectively, and were composed of 7 regions of interest for the auditory network, 9 for the DMN and 11 for the FPN (coordinates are specified in Supplementary Material VI) (Demertzi *et al.*, 2015; Aubinet *et al.*, 2018). At second level, we focused on positive connectivity and performed one-sided test difference contrasts between the patient groups (UWS LOCA, UWS NO-LOCA and MCS−) and HCS but also between the two groups of UWS (LOCA and NO-LOCA), and between the two groups of UWS and the group of MCS−. The standardized age and the time since injury (log-value) were both taken as covariates. The results were considered statistically significant at the cluster-wise threshold *P* < 0.05 FDR corrected for multiple comparison at non-parametric permutation test cluster-mass (Chumbley *et al.*, 2010; Pernet *et al.*, 2015) and using a voxel-wise *P*-uncorrected <0.001 (whole-brain level) cluster-forming threshold, as implemented in standard CONN.

#### High-density electroencephalography

Finally, we collected resting-state high-density EEG (hd-EEG) recordings with 256 channels (Electric Geodesics system, EGI) for ∼20 min with a sampling rate of 500 Hz. Electroencephalography data were preprocessed as described elsewhere (Supplementary Material V) (Chennu *et al.*, 2017). Electroencephalography power spectra were then decomposed into delta (1–4 Hz), theta (4.1–8 Hz), alpha (8.1–12 Hz) and beta (12.1–30 Hz) bands, and analysed with multi-taper method (Percival and Walden, 1993). Mean powers of whole brain and frontal, parietal, central, temporal, occipital, upper and lower midline brain areas (Supplementary Material V) were estimated for each frequency band and for each group [LOCA, NO-LOCA, MCS− and 26 HCS (mean age, 44 ± 16 years, 14 women)]. Mean connectivity of whole brain and separated brain regions was also measured for all frequency bands in each group using debiased weighted Phase Lag Index, as described elsewhere (Chennu *et al.*, 2017). Brain network topological properties of whole brain and separated brain regions were subsequently measured using graph theory measures of network centrality and summarized by the deviation of the participation coefficient (Chennu *et al.*, 2017).

Statistical analyses were performed using non-parametric multivariate permutation test (Pierezan, 2019) (5000 permutations) with MATLAB 2018a, to test group differences in terms of connectivity and graph theory measures. The standardized age and the time since injury were taken as factors, along with connectivity and graph theory measures in the multivariate permutation test. Brain region-wise EEG measures were computed by taking region-wise averages of the per-electrode values of power, connectivity and graph-theory metrics. Multiple comparisons corrections were carried out over the range of brain regions (*n* = 12) using FDR correction *P* < 0.05.

Patients underwent FDG-PET, fMRI and/or hdEEG recordings within a maximum of 10 days.

### Data availability

The data that support the findings of this study are available from the corresponding author, upon reasonable request.

## Results

### Participants

Demographic data of the whole sample of patients and of each paraclinical assessment for the UWS LOCA and UWS NO-LOCA are reported in Table 1.

**Table 1:** Demographic data summary of the large sample of patients (*n* = 186) and comparison of UWS patient subgroups (LOCA and NO-LOCA) according to demographic data

	Behavioural					^18^F-FDG-PET			fMRI			hdEEG		
	Whole sample	UWS	MCS−	MCS+	EMCS	UWS NO-LOCA	UWS LOCA	*P*-value	UWS NO-LOCA	UWS LOCA	*P*-value	UWS NO-LOCA	UWS LOCA	*P*-value
No. of participants	186	64	28	71	23	30	8		25	8		15	5	
Mean age ± SD (median; range)	39 ± 16 (37; 16–79)	42 ± 16 (39; 16–77)	39 ± 13 (37.5; 19–66)	40 ± 17 (36; 18–79)	40 ± 15 (30; 18–66)	46 ± 14 (45; 20–74)	32 ± 9 (31.5; 21–46)	***P*** **=** 0.014^*^	47 ± 15 (44; 20–74)	32 ± 9 (31.5; 21–46)	***P*** **=** 0.019^*^	45 ± 15 (39; 20–73)	28 ± 7 (26; 21–40)	***P*** **=** 0.038^*^
Gender (women/men)	66/120	25/39	9/19	24/47	7/16	17/13	3/5	*P* = 0.438	13/12	3/5	*P* = 0.688	5/10	2/3	*P* = 1.000
Aetiology (TBI/NTBI)	100/86	20/44	25/13	48/23	16/7	6/24	3/5	*P* = 0.363	5/25	3/5	*P* = 0.366	2/13	2/3	*P* = 0.249
Median time since injury in months (min–max)	9 (1–359)	9 (1–228)	10 (1–359)	17 (1–297)	12 (1–240)	6 (1–66)	19 (2–168)	***P*** **=** 0.017^*^	6 (1–66)	19 (2–168)	***P*** **=** 0.009^*^	14 (1–359)	15 (9–168)	*P* = 0.541

FDG-PET, fluorodeoxyglucose positron emission tomography; fMRI, functional magnetic resonance imaging; hdEEG, high-density electroencephalography; SD, standard deviation; TBI, traumatic brain injury; NTBI, non-traumatic brain injury.

* **Statistically significant (*P*** **<** **0.05).**

Age did not significantly differ between UWS LOCA, UWS NO-LOCA and HCS (FDG-PET: *P* = 0.452; fMRI: *P* = 0.128; hdEEG: *P* = 0.113), neither did gender (FDG-PET: *P* = 0.562; fMRI: *P* = 0.493; hdEEG: *P* = 0.431). For UWS patients, LOCA and NO-LOCA groups did not significantly differ in terms of gender or aetiology. The LOCA group had, however, a lower age and the time since injury was longer (except for the EEG group) (Table 1).

Demographic data of the subgroup of MCS− patients and their comparison with UWS LOCA and NO-LOCA are reported in Supplementary Material III. The list of the pharmacological agents acting on the nervous system can be found for the patients who underwent neuroimaging/electrophysiological examinations in Supplementary Material XI.

### Behavioural and outcome data

**Figure 2:**
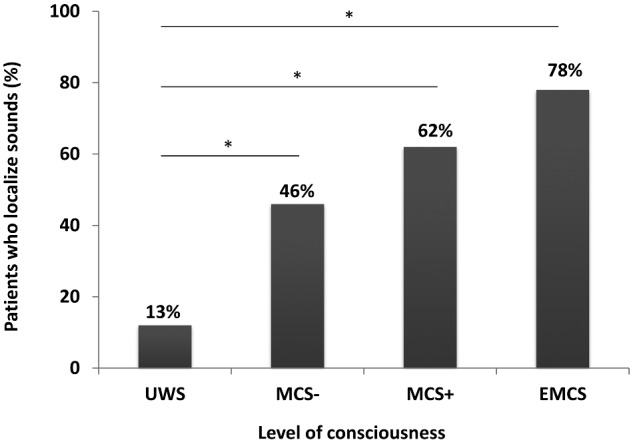
**Behavioural results.** Proportion of auditory localization among post-comatose patients. A relationship was observed between the presence of auditory localization and the level of consciousness (χ^2^ = 45.94, d*f* = 3, *P* < 0.001). A significant difference was found between UWS and MCS−, UWS and MCS+, and UWS and EMCS. Abbreviations: UWS = unresponsive wakefulness syndrome; MCS− = minimally conscious state minus; MCS+ = minimally conscious state plus; EMCS = emergence from the minimally conscious state. *P < 0.001.

Auditory localization was present in 83 out of 186 patients (45%): 8 out of 64 patients in UWS (13%), 13 out of 28 patients in MCS− (46%), 44 out of 71 patients in MCS+ (62%) and 18 out of 23 patients in EMCS (78%) (Fig. 2). A positive relationship was observed between the presence of auditory localization and the level of consciousness (χ^2^(3) = 45.94, *P* < 0.001, φ = 0.497). The probability to present an auditory localization increases along the level of consciousness. Chi-square tests provided evidence of a significant difference for the presence of auditory localization between UWS and MCS− (χ^2^(1) = 10.875, *P *= 0.001, φ = 0.372), UWS and MCS+ (χ^2^(1) = 32.729, *P* < 0.001, φ = 0.508), and UWS and EMCS patients (χ^2^(1) = 31.851, *P* < 0.001, φ = 0.634). There was also a greater proportion of TBI patients who showed auditory localization (53%) compared to non-TBI patients (35%) (χ^2^(1) = 5.430, *P* = 0.018, φ = −0.182).

Among patients with auditory localization, groups differed in terms of repeatability, with a median repeatability percentage of 29% in UWS, 50% in MCS−, 39% in MCS+ and 53% in EMCS patients (H(3) = 8.191, *P* = 0.042). For the patients without auditory localization, auditory startle was observed in 49/55 UWS (89%), 12/15 MCS− (80%), 21/27 MCS+ (78%) and 5/5 EMCS (100%) patients, without significant difference between groups (χ^2^(3) = 3.045, *P* = 0.385, φ = −0.173).

Finally, we employed a multinomial logistic regression to predict the level of consciousness based on the clinical data. The model was statistically significant (χ^2^(12) = 66.105, *P* < 0.001, *R*^2^ Cox and Snell = 0.299). The aetiology (*P* = 0.004) and auditory localization (*P* < 0.001) significantly predicted the level of consciousness, but not the age (*P* = 0.780) nor the time since injury (*P* = 0.756).

Table 2 reports the clinical data of the eight UWS patients with LOCA. It should be noted that four out of eight show atypical behaviours, such as swallowing or resistance to eye opening (Mélotte *et al.*, 2018, 2020; van Ommen *et al.*, 2018).

**Table 2:** Demographical, clinical and outcome data of the eight UWS patients with auditory localization

Patient	Age and gender	Aetiology	Time since injury (months)	Best CRS-R sub-scores	Best CRS-R total score	Occurrence of auditory localization	Atypical behaviours^a^	Outcome at 2 years
UWS_LOCA01	41 M	CA	26	Auditory: localization Visual: none Motor: reflex Oromotor/verbal: reflex Communication: none Arousal: with stimulation	5	1/5	None	GOSE = 3
UWS_LOCA02	21 F	TBI	9	Auditory: localization Visual: none Motor: reflex Oromotor/verbal: reflex Communication: none Arousal: without stimulation	6	1/5	None	GOSE = 1
UWS_LOCA03	32 M	CA	168	Auditory: startle^a^ Visual: blinking to threat Motor: localization Oromotor/verbal: reflex Communication: none Arousal: without stimulation	7	1/5	Orally fed (liquids and semi-liquids)	GOSE = 2
UWS_LOCA04	40 F	CA	24	Auditory: localization Visual: none Motor: reflex Oromotor/verbal: reflex Communication: none Arousal: with stimulation	5	1/5	None	Missing outcome
UWS_LOCA05	26 M	TBI	15	Auditory: localization Visual: blinking to threat Motor: localization Oromotor/verbal: reflex Communication: none Arousal: with stimulation	7	1/5	Operational swallowing with creams and liquids, resistance to eye opening	GOSE = 3
UWS_LOCA06	46 F	CA	2	Auditory: localization Visual: none Motor: none Oromotor/verbal: none Communication: none Arousal: with stimulation	3	1/5	None	GOSE = 1
UWS_LOCA07	31 M	TBI	36	Auditory: localization Visual: none Motor: localization Oromotor/verbal: reflex Communication: none Arousal: with stimulation	6	4/5	None	GOSE = 1
UWS_LOCA08	23 M	Anoxia	15	Auditory: startle^b^ Visual: blinking to threat Motor: localization Oromotor/verbal: none Communication: none Arousal: without stimulation	8	2/6	Legs crossing, operational swallowing with creams and liquids	GOSE = 2

UWS_LOCA, UWS patients with auditory localization; M, male; F, female; CA, cardiac arrest; TBI, traumatic brain injury; CRS-R, Coma Recovery Scale Revised; GOSE, Glasgow Outcome Scale Extended.

a Behaviours that have been associated with consciousness or with a favourable outcome in recent literature [swallowing (Mélotte *et al.*, 2018, 2020), resistance to eye opening (van Ommen *et al.*, 2018) and legs crossing (Rémi *et al.*, 2011)].

b UWS_LOCA03 and UWS_LOCA08 did not show auditory localization at the time of the CRS-R with the best score.

Outcome data were available for 125 out of 186 patients (67%). At the whole sample (*n* = 125), a difference in the survival rate was found between patients with and without auditory localization, with 80% (43/54) of patients with auditory localization still alive compared to 55% (39/71) of patients who did not show any auditory localization (χ^2^(1)=8.29, *P* = 0.002, φ = 0.26). To limit the variability of the time since injury, we performed the same analysis on a subsample (*n* = 95) of patients who were <3 years of post-injury and we found similar results (χ^2^(1) = 6.958, *P *= 0.010, φ = 0.27). The details of the outcome of the whole sample of patients are available in Supplementary Material VII. Looking only at UWS patients, 29% (2/7) of LOCA patients recovered some signs of consciousness compared to 8% (3/38) of NO-LOCA patients (GOSE = 3), with 57% (4/7) patients alive after 2 years in the LOCA group compared to 42% (16/38) in the NO-LOCA group. No significant difference was found in the recovery of consciousness (χ^2^(1) = 2.559, *P* = 0.167, φ = 0.239) and in the survival rate (χ^2^(1) = 0.117, *P* = 1.00, φ= 0.055) in the UWS group.

### Brain imaging in UWS LOCA and NO-LOCA patients

#### [^18^F]-fluorodeoxyglucose positron emission tomography

**Figure 3:**
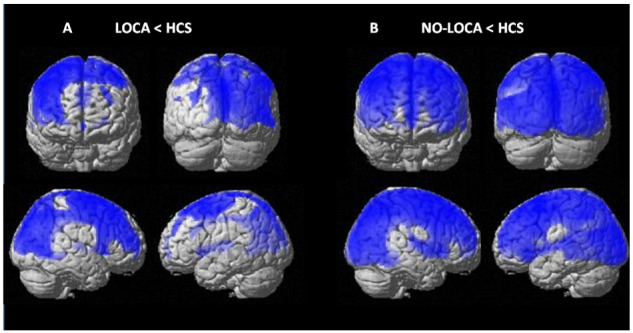
**^18^FDG-PET results.** Areas showing significant metabolic impairment (in blue) in **A** NO-LOCA and **B** LOCA patients in UWS compared with HCS, thresholded at *P* < 0.05 FDR-corrected for multiple comparisons (at the whole-brain level).

NO-LOCA patients showed decreased metabolism in a large bilateral fronto-parieto-occipital network, in particular, in the right ventral posterior cingulate cortex, left premotor cortex, left angular gyrus, left sensorimotor associative regions, bilateral frontal eye fields and thalamus (Fig. 3 and Supplementary Material VIII). LOCA patients showed regional decreased metabolism (compared to HCS), with the hotspots located in the ventral anterior and posterior cingulate cortex, left premotor cortex, right frontal eye fields, right angular gyrus, right visual secondary and associative areas and right fusiform gyrus. The direct comparison between UWS LOCA and NO-LOCA did not show any statistical difference.

#### Functional MRI

**Figure 4:**
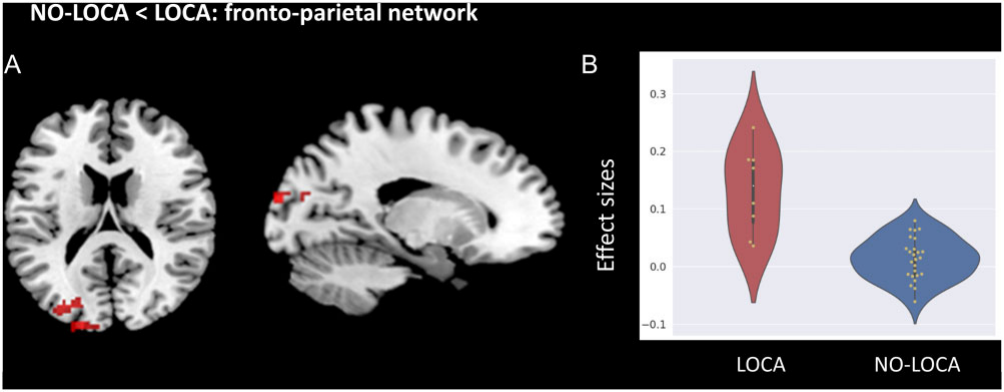
**fMRI results.** (**A**) Brain areas showing higher functional connectivity in LOCA compared to NO-LOCA patients in UWS for the fronto-parietal network. Statistical maps are thresholded at *P* < 0.05 FDR-corrected at non-parametric cluster-mass, with clusters made of voxels surviving a *P* < 0.001 uncorrected (whole-brain level). (**B**) Effect sizes (*z*-values) for the cluster showing higher connectivity in LOCA (in red) as compared to NO-LOCA (in blue) for the fronto-parietal network.

For the FPN, NO-LOCA patients showed less connectivity compared to HCS between the FPN and four clusters covering (i) the temporo-occipital part of the middle temporal gyrus, (ii) the angular gyrus, (iii) the right thalamus and (iv) the posterior division of the supramarginal gyrus and the superior division of the lateral occipital cortex. The LOCA patients showed less connectivity compared to HCS between this network and five clusters covering (i) the posterior division of the middle temporal gyrus, (ii) the occipital fusiform gyrus, (iii) the temporo-occipital part of the middle temporal gyrus, (iv) the superior frontal gyrus and (v) the parietal regions (angular and supramarginal gyri) (Supplementary Materials IX and X). The LOCA patients had higher connectivity between the FPN and a cluster, covering the occipital pole and the lateral occipital cortex compared to NO-LOCA patients (Fig. 4A). The *Z*-scores of the effect sizes for this occipital cluster resulting from the LOCA>NO-LOCA contrast are shown in Fig. 4B.

For the auditory network, NO-LOCA patients showed less connectivity compared to HCS between the auditory network and six clusters covering (i) the superior division of the right lateral occipital cortex, the cuneal cortex and lingual gyrus, (ii) the right insular cortex, planum polare and post-central gyrus, (iii) the left planum polare, precentral gyrus and planum temporale, (iv) the left lingual gyrus, (v) the right post-central gyrus, supplementary motor cortex and anterior cingulate gyrus and (vi) the left lateral occipital cortex. The LOCA patients also showed less connectivity compared to HCS between this network and seven clusters covering (i) the anterior division of the right superior temporal gyrus, the right precentral gyrus and the insular cortex, (ii) the left precentral gyrus and planum temporale, (iii) the supplementary motor cortex, (iv) the right precentral gyrus, (v) the right cuneal cortex, (vi) the right lingual cortex and (vii) the right pre- and post-central gyri (Supplementary Materials IX and X). No difference was found between LOCA and NO-LOCA patients.

For the DMN, NO-LOCA patients showed less connectivity compared to HCS between the DMN and eight clusters covering (i) the posterior cingulate gyrus and precuneus, (ii) the left superior lateral occipital cortex and angular gyrus, (iii) the paracingulate gyrus and the right superior frontal gyrus, (iv) the right angular gyrus and superior lateral occipital cortex, (v) the left middle frontal gyrus, (vi) the left temporal pole, (vii) the right anterior middle temporal gyrus and temporal pole and (viii) the left posterior parahippocampal gyrus. The LOCA patients showed less connectivity compared to HCS between this network and four clusters covering (i) the precuneus, (ii) the left superior lateral occipital cortex, (iii) the anterior cingulate and paracingulate gyri and (iv) the right angular gyrus and superior lateral occipital cortex (Supplementary Materials IX and X). No difference was found between LOCA and NO-LOCA patients.

### High-density electroencephalography

**Figure 5:**
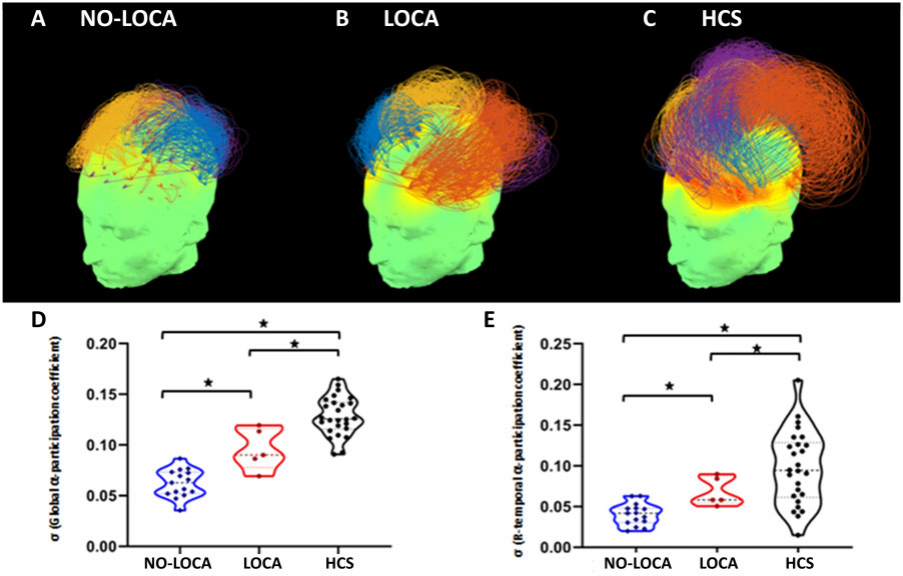
**EEG results.** Alpha band connectivity topographs in **A** NO-LOCA patients in UWS, **B** LOCA patients in UWS and **C** HCS. Connectivity is demonstrated in the 3D scalp topography, the colour map over the scalp shows degrees (total connection) of nodes in the network. Arcs connect pairs of nodes, and their normalized heights indicate the strength of connectivity between them. The colour of an arc indicates the network module to which it belongs. (**D**) Graph theory measures of participation coefficient showed a significant difference in alpha frequency band between LOCA (in red) and NO-LOCA (in blue) patients in UWS in the whole brain (*P* = 0.002) and **E** in right temporal regions (*P* = 0.002). The stars indicate significant (*P* < 0.05) participation coefficients.

Power spectral measures and mean connectivity did not differ between the two groups of patients. Graph theoretic analysis of alpha band hd-EEG networks indicated stronger connectivity between frontal and parietal electrodes in LOCA compared to NO-LOCA patients. This phenomenon is shown in Fig. 5, with topological modules consisting of coloured connections between these electrodes. Going from NO-LOCA (a) and LOCA (b) to HCS (c), these modules were progressively stronger and spanned greater topographical distance. Quantitative analysis of these networks identified significantly higher standard deviation in participation coefficients—indexing network centrality—in the alpha band network of LOCA compared to NO-LOCA patients at the whole-brain level (Fig. 5D). Supplementary brain region-wise analysis confirmed that this difference was also significant in right temporal regions (Fig. 5E). Besides alpha, we did not find any other significant differences in the other frequency bands.

### Comparison with MCS− patients

**Figure 6:**
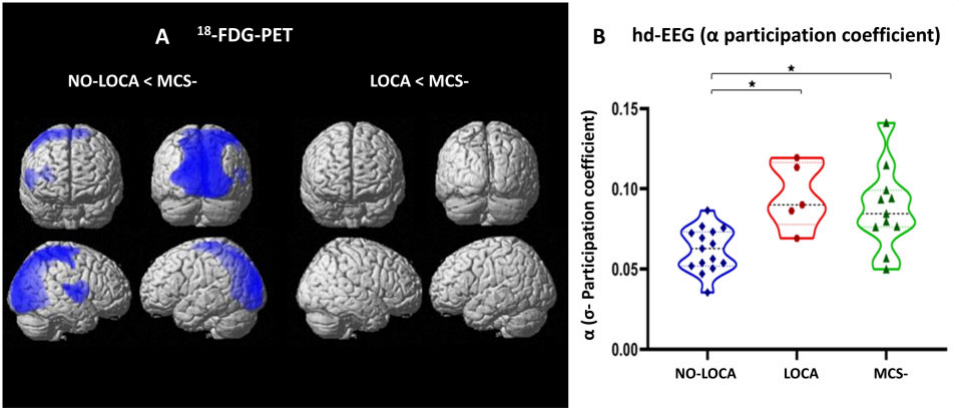
**Results of ^18^FDG-PET and hd-EEG analyses comparing UWS LOCA and NO-LOCA patients with patients in MCS minus.** (**A**) With ^18^FDG-PET, we observed more hypometabolism (blue spots) in NO-LOCA compared to MCS minus patients, whereas there was no significant difference between LOCA and MCS minus patients. (**B**) Hd-EEG analyses revealed a significant difference in alpha participation coefficient between NO-LOCA and MCS minus patients (*P* = 0.001), but not between LOCA and MCS minus patients.

No significant difference in brain metabolism was found between UWS LOCA patients and MCS− patients. Compared to MCS− patients, UWS NO-LOCA patients showed decreased hypometabolism in the bilateral visual secondary cortex, right primary auditory cortex, right precuneus, right primary somatosensory cortex, bilateral primary motor cortex, right premotor cortex and right visual associative cortex (Fig. 6A). No higher functional connectivity was found in MCS− compared to UWS LOCA and NO-LOCA patients, for any of the three fMRI networks. In hdEEG, we did not find any significant difference in network centrality (measured by the standard deviation of alpha participation as described above) between UWS LOCA and MCS− patients, but a significant difference was found between UWS NO-LOCA and MCS− patients at the whole-brain level (*P* = 0.001, Fig. 6B), at the lower midline (*P* = 0.001) and in the right temporal regions (*P* = 0.007).

## Discussion

In the current literature, there is no agreement whether auditory localization constitutes a purely reflex or higher-order behaviour. In the CRS-R, which is the recommended scale to assess the level of consciousness of post-comatose patients, auditory localization is not included in the MCS criteria. We here aimed to test the hypothesis that auditory localization is a conscious behaviour and reflects a higher-level cognitive processing, using a multimodal approach. As expected, we found that the probability to observe an auditory localization increases along with the level of consciousness. Although we did not find any differences in terms of clinical improvement at 2-year follow-up, we found a higher survival rate in patients showing auditory localization compared to those who did not (regardless of their diagnosis). At the brain level, FDG-PET analysis did not reveal significant differences between UWS LOCA and NO-LOCA patients. We, however, found as hypothesized higher functional connectivity in brain regions supporting awareness in UWS LOCA patients compared to UWS NO-LOCA patients, as shown by MRI and EEG. Patients with UWS LOCA patients also show brain similarities with MCS− patients, compared to UWS NO-LOCA. Overall, these clinical and brain imaging findings support our initial hypothesis that auditory localization may reflect a higher-level processing and corresponds to an MCS rather than an UWS criteria.

Clinically, we showed that the probability to observe an auditory localization increases along with the level of consciousness and that the presence or absence of auditory localization can significantly predict the level of consciousness. Moreover, among the patients without auditory localization, the great majority of them presented an auditory startle, ruling out the hypothesis of deafness. For the remaining patients, vigilance fluctuations and severe motor deficits (including oculomotor paralysis and supranuclear ocular motor damage) might explain the absence of auditory response. We also showed that the proportion of patients with auditory localization is higher in TBI than non-TBI patients, which could be explained by more widespread and diffuse lesions usually observed in non-TBI etiologies. Although we did not find any differences in terms of clinical improvement at 2-year follow-up, we found a higher survival rate in patients showing auditory localization compared to those who did not.

At the brain level, FDG-PET analysis did not reveal significant differences between UWS LOCA and NO-LOCA patients. However, fMRI analyses highlighted higher functional connectivity between the FPN (linked to external awareness) and occipital regions (Brodmann areas 18 and 19), in LOCA compared to NO-LOCA patients. Although we could have expected to find higher connectivity in auditory-related regions in UWS LOCA, it is important to remind that these fMRI results do not reflect network connectivity during an auditory localization task, but at rest. Consequently, UWS LOCA and NO LOCA do not seem to differ in terms of connectivity in auditory-related brain regions, but rather in consciousness-related brain regions, and particularly those linked to external awareness such as the FPN. The observation of higher functional connectivity in the FPN is not surprising, given that auditory localization is a behaviour observed after external stimulation, and that should therefore imply external awareness (rather than internal awareness). The FPN has been identified as part of the external network because of its connections with the sensory subsystems (Golland *et al.*, 2007), and it has been linked to cognitive processes of somatosensory (Bornhövd *et al.*, 2002; Boly *et al.*, 2007), visual (Dehaene and Changeux, 2011) and auditory (Brunetti *et al.*, 2008) sensory inputs. Interestingly, the two occipital regions (also referred to as the extrastriate or secondary visual cortex) with higher connectivity with the FPN were found to be hypometabolic in UWS NO-LOCA compared to MCS− patients in our ^18^FDG-PET analysis. In blind subjects, the involvement of occipital regions in auditory processing is well-established (Alho *et al.*, 1993; Gougoux *et al.*, 2005; Voss *et al.*, 2006; Stevens *et al.*, 2007), but a role of the visual cortex in spatial hearing and active listening has been suggested by several studies in sighted subjects as well (Zimmer *et al.*, 2004; Lewald *et al.*, 2004a; Carmody and Lewis, 2006; Degerman *et al.*, 2006; Wu *et al.*, 2007; Cate *et al.*, 2009). Overall, these studies suggest that the involvement of secondary visual areas in the processing of sounds might therefore help to explain the stronger connectivity observed in UWS LOCA compared to NO-LOCA patients.

Regarding hdEEG, results indicated higher participation coefficient in the alpha frequency band in the whole brain and in the right temporal regions in LOCA compared to NO-LOCA patients. The fact that we obtained a difference between our two UWS groups only for the alpha band is consistent with the existing literature, stating that only the graph-theory metrics of this frequency band correlate with the degree of consciousness (Chennu *et al.*, 2014, 2017). Besides, the participation coefficient in the alpha band is one of the most effective for discriminating UWS from MCS patients (Chennu *et al.*, 2014, 2017), therefore providing additional evidence in favour of our hypothesis that UWS patients showing auditory localization should be considered as MCS− patients. As regards to right temporal regions, both functional neuroimaging and lesion studies support the implication of these areas in a variety of tasks involving pitch processing (Zatorre and Samson, 1991; Zatorre *et al.*, 1992)*.* The right temporal cortex was also found to be responsible for the processing of acoustic properties of voices (Kriegstein and Giraud, 2004) and emotional prosody (Mitchell *et al.*, 2003; Ethofer *et al.*, 2006).

Additionally, when comparing with MCS− patients, UWS NO-LOCA patients presented a significantly decreased metabolism in a large parieto-occipital network, whereas there was no statistically significant difference between MCS− and UWS LOCA patients. The UWS LOCA patients were also more similar to MCS− patients using the participation coefficient of the alpha band, the values of this metric for LOCA patients being in the range of those of the MCS− patients.

All these results suggest that auditory localization should be considered as a more complex behaviour than a simple reflex, therefore reflecting higher cognitive processes. If these findings are confirmed in a further study, patients diagnosed as UWS who present auditory localization as defined in the CRS-R guidelines could therefore be considered as in MCS. Indeed, while some auditory behaviours may rely mostly on brainstem structures, others may relate to a richer cognitive state. Consequently, a new semiology of auditory behaviours should be investigated to provide more nuanced criteria taking into account different types of responses, as Hermann *et al.* (2020) recently did by proposing habituation of auditory startle reflex as a sign of MCS. Finally, the clinical picture of auditory localization without the presence of any other MCS items should prompt examiners to consider the presence of visual impairments or motor disorders/spasticity that would prevent patients from displaying MCS items in the other CRS-R subscales (Chatelle *et al.*, 2016).

Several limitations should be acknowledged. The main one is the small size (*n* = 8) of our sample of UWS LOCA patients, which is due to the scarcity of this specific clinical picture (i.e. the presence of auditory localization without any other sign of consciousness at bedside). Indeed, MCS patients showing only one sign of consciousness are not so frequent (Wannez *et al.*, 2018). The fact that the different imaging modalities generated convergent evidence in the small sample is, however, a reassuring argument and we appropriately used non-parametric statistics to alleviate the challenge of invalid parametric assumptions in small samples. Another limitation is that the subgroups of patients did not match for age and time since injury. Time since injury was variable across patients due to our clinical setting, and to ensure that the results were not driven by the effect of these confounding factors, age and time since injury were both taken as covariates in MRI and EEG analyses. Moreover, a difference in the survival rate between patients with and without auditory localization was also found in a subsample of patients who were <3 years post-injury. One should yet note the number of missing clinical outcome data in our initial sample, which are difficult to obtain in this challenging population. Future research documenting the incidence of auditory localization in the (sub)acute setting and comparing the evolution of patients who showed (or not) auditory localization in the early stage after recovering from coma is definitely needed. Another potential limitation is that some patients received light sedation during fMRI acquisition because of movement artefact. Yet, the group of LOCA patients still showed higher connectivity with the FPN than the other group, although it included a larger proportion of sedated patients (75% versus 36% of NO-LOCA patients). This suggests that sedation did not have a major impact on our results, or if any, it provided an underestimation of the difference between LOCA and NO-LOCA. Future studies should also look at auditory evoked potentials (e.g. mismatch negativity and P300) or otoacoustic emissions, which would provide additional information on auditory processing. Without those additional exams, we cannot here rule out deafness in the complete absence of auditory response.

## Conclusion

In conclusion, our multimodal results converge towards our initial hypothesis that auditory localization should be considered as a new sign of MCS. We found that the probability to observe an auditory localization increases along with the level of consciousness. Besides, patients with auditory localization have better survival rates. We also showed differences in brain functioning between the two subgroups of UWS patients, with LOCA patients being more similar to MCS− patients. If our findings are confirmed by future studies with larger samples, UWS patients showing auditory localization (UWS LOCA) should therefore no longer be considered in UWS but in MCS−. Even if the presence of auditory localization concerns only a minority of UWS patients (in our sample 13%), it would have crucial consequences on these patients’ lives and their relatives to consider this behaviour as conscious, given that important decisions regarding treatment (e.g. pain), therapeutic orientation (e.g. rehabilitation), but also end-of-life decisions are frequently taken based on the diagnosis.

## Supplementary material

Supplementary material is available at *Brain Communications* online.

## Supplementary Material

fcaa195_Supplementary_Data

## Abbreviations

CRS-R =: Coma Recovery Scale-Revised
DMN =: default mode network
DOC =: disorders of consciousness
EMCS =: emergence from the minimally conscious state
^18^FDG-PET =: [^18^F]-fluorodeoxyglucose positron emission tomography
FPN =: fronto-parietal network
FDR =: False-discovery rate
GOSE =: Glasgow Outcome Scale-Extended
HCS =: healthy control subjects
hdEEG =: high-density electroencephalography
LOCA =: patients who present auditory localization
MCS− =: minimally conscious state minus
MCS+ =: minimally conscious state plus
NO-LOCA =: patients without auditory localization
UWS =: unresponsive wakefulness syndrome
