# Supplementary material for: Auditory localization should be considered as a sign of minimally conscious state based on multimodal findings
Source: Brain Commun. 2020 Dec 12;2(2):fcaa195. doi: 10.1093/braincomms/fcaa195 (PMC7784043; doi:10.1093/braincomms/fcaa195)
Supplement: fcaa195_Supplementary_Data [file fcaa195_supplementary_data.pdf]

Online supplementary appendix to:

Auditory localisation should be considered as a sign of minimally conscious state based on multimodal findings

Carrière Manon[1,2], MSc, Cassol Helena[1,2], MSc, Aubinet Charlène[1,2], MSc, Panda Rajanikant[1,2], MSc, Thibaut Aurore[1,2], PhD, Larroque Stephen K.[1,2], MSc, Simon Jessica[3], PhD, Martial Charlotte[1,2], PhD, Bahri Mohamed A.[4], PhD, Chatelle Camille[1,2], PhD, Martens Géraldine[1,2], MSc, Chennu Srivas[5,6], PhD, Laureys Steven[1,2], PhD & Gosseries Olivia[1,2], PhD.

[1] Centre du Cerveau[2], University Hospital of Liège, Liège, Belgium.
[2] Coma Science Group, GIGA-Consciousness, University of Liège, Liège, Belgium
[3] Psychology and Neurosciences of Cognition Research Unit, University of Liège, Belgium
[4] GIGA-Cyclotron Research Centre in Vivo Imaging, University of Liège, Liège, Belgium
[5] School of Computing, University of Kent, UK
[6] Department of Clinical Neurosciences, University of Cambridge, UK

This document includes:

Supplementary I. Inclusion criteria
Supplementary II. Demographic and clinical data of patients in UWS with at least one paraclinical assessment.
Supplementary III. Demographic data summary of MCS- patients and comparison with UWS LOCA and NO-LOCA patients.
Supplementary IV. Behavioral acquisitions using the Coma Recovery Scale-Revised.
Supplementary V. $^{18(}$F-)FDG-PET, MRI and EEG data acquisition parameters, preprocessing and analysis.
Supplementary VI. Seeds coordinates used in fMRI analyses, for the auditory, default mode and fronto-parietal networks.
Supplementary VII. Outcome data of the whole sample of patients (n=186)
Supplementary VIII. ^{18}F-FDG-PET results: statistical values of the clusters obtained by the direct comparisons of LOCA and NO-LOCA patients with HCS.
Supplementary IX. fMRI results: Comparison between LOCA and HCS (on the left) and between NO-LOCA and HCS (on the right) of the correlation between the auditory (first row), default mode (second row) and fronto-parietal (third row) networks and the time series from all other brain voxels. Statistical maps are thresholded at p<0.05 false discovery rate corrected at non-parametric cluster-mass with clusters made of voxels surviving a p<0.001 (whole-brain level).
Supplementary X. fMRI results: statistical values of the clusters obtained by the comparisons of LOCA patients and HCS, and NO-LOCA patients and HCS for the auditory, default mode and fronto-parietal networks, and the comparison of LOCA and NO-LOCA, for the fronto-parietal network only.
Supplementary XI. List of all pharmacological agents acting on the nervous system, taken by the two groups of UWS patients (LOCA and NO-LOCA).

I. Inclusion criteria.

Patients were admitted to the Hospital University of Liege in Belgium between 2010 and 2018 for a multimodal assessment of the level of consciousness and they were included in the study if the following criteria were met:

- ≥18 years old
- > 28 days post-injury (prolonged DOC)
- At least five repeated assessments with the Coma Recovery Scale-Revised
- Diagnosis of UWS, MCS or EMCS
- Absence of previous neurological or psychiatric conditions

II. Demographic and clinical data of patients in UWS and MCS minus with at least one paraclinical assessment.

Patient	Gender	Age	Etiology	Time since injury	Best CRS-R total score	Outcome at 2 years post-evaluation (GOSE)	fMRI	^{18}F-FDG-PET	hd-EEG	Sedation for fMRI
UWS-loca1	M	41	CA	2 years	5	3	X	X		Yes
UWS-loca2	F	21	TBI	9 months	5	1	X	X	X	No
UWS-loca3	M	32	CA	14 years	7	2	X	X	X	Yes
UWS-loca4	F	40	CA	2 years	5		X	X	X	Yes
UWS-loca5	M	26	TBI	15 months	7	3	X	X	X	Yes
UWS-loca6	F	46	CA	2 months	3	1	X	X		Yes
UWS-loca7	M	31	TBI	3 years	5	1	X	X		No
UWS-loca8	M	23	Anoxia	15 months	7	2	X	X	X	Yes
UWS-noloca1	F	53	Hemorrhage	1 month	5	1	X	X		No
UWS-noloca2	F	46	Anoxia	6 months	6	1	X	X		No
UWS-noloca3	M	56	Anoxia	8 months	5	2		X		
UWS-noloca4	F	48	Anoxia	1 year	6	1	X	X		No
UWS-noloca5	F	36	Anoxia	4 years	5	1		X	X	
UWS-noloca6	M	43	Anoxia	1 month	5	1	X	X		No
UWS-noloca7	M	67	CA	1 month	3	1	X	X		No
UWS-noloca8	M	43	Anoxia	2 months	5	1	X	X		No
UWS-noloca9	M	56	CA	6 months	6	1	X	X	X	No
UWS-noloca10	M	53	Meningitis	2 months	5	1	X	X		Yes
UWS-noloca11	F	34	SAH	1 year	5		X	X	X	Yes
UWS-noloca12	F	24	TBI	2 years	6	1	X	X		No
UWS-noloca13	M	20	TBI	10 months	6		X	X	X	Yes
UWS-noloca14	F	59	CA	3 years	6	1		X	X	
UWS-noloca15	F	73	Stroke	1 month	7	1	X	X		No
UWS-noloca16	F	68	Anoxia	1 month	5		X	X		No
UWS-noloca17	F	68	Anoxia	1 year	6	1	X	X		Yes
UWS-noloca18	F	65	TBI	7 months	7	1	X	X		No
UWS-noloca19	F	74	CA	1 month	5	1	X	X		Yes
UWS-noloca20	F	48	CA	4 months	4	1	X	X		No
UWS-noloca21	M	39	TBI	1 year	5	1	X	X		No
UWS-noloca22	F	47	CA	6 months	5	1		X	X	
UWS-noloca23	M	41	Anoxia	11 months	5	2	X	X		Yes
UWS-noloca24	F	31	TBI	13 months	6	3		X		
UWS-noloca25	M	30	Anoxia	5 years	6	2	X	X	X	No
UWS-noloca26	F	38	Anoxia	11 months	5	2	X	X	X	No
UWS-noloca27	M	39	TBI	13 months	5	1	X	X	X	Yes
UWS-noloca28	F	44	Anoxia	3 months	6	1	X	X		Yes
UWS-noloca29	M	31	Anoxia	15 months	6		X	X		Yes
UWS-noloca30	M	28	Anoxia	2 years	7	2	X	X	X	No
UWS-noloca31	M	73	CA	3 months	6	1			X	
UWS-noloca32	M	35	CA	19 years	7	1			X	
UWS-noloca33	M	65	Anoxia	5 months	6	1			X	
UWS-noloca34	M	62	CA	2 years	5	1			X	
UWS-noloca35	M	49	Anoxia	8 years	6	1			X	
MCSminus1	F	40	Stroke	7 months	9		X	X	X	Yes
MCSminus2	F	49	TBI	7 months	7	3	X	X		Yes
MCSminus3	M	26	TBI	17 months	8		X	X		Yes
MCSminus4	M	47	Anoxia	4 years	13	3	X	X		Yes
MCSminus5	F	59	Anoxia	1 month	9	1		X	X	
MCSminus6	F	26	CA	2 years	7	2	X	X		Yes
MCSminus7	M	28	TBI	2 months	7	2	X	X	X	Yes
MCSminus8	M	21	TBI	6 months	7	2	X	X	X	Yes
MCSminus9	M	53	Anoxia	1 month	15	1		X		
MCSminus10	M	24	TBI	11 months	10	3	X	X	X	No
MCSminus11	F	36	TBI	4 years	10	2	X	X	X	No
MCSminus12	M	19	TBI	3 years	9			X	X	
MCSminus13	F	47	Anoxia	15 months	7	3	X	X	X	Yes
MCSminus14	M	41	TBI	29 years	14			X		
MCSminus15	F	45	TBI	8 months	7	1	X	X		No
MCSminus16	M	22	TBI	8 years	13	3	X	X	X	Yes
MCSminus17	M	60	TBI	5 years	13			X		
MCSminus18	F	24	TBI	11 months	10		X	X	X	Yes
MCSminus19	M	53	Anoxia	5 months	13		X	X		Yes
MCSminus20	M	30	CA	20 months	13	3	X			Yes
MCSminus21	M	66	Stroke	1 month	12		X			No
MCSminus22	M	28	TBI	7 months	10		X			Yes
MCSminus23	M	49	Anoxia	3 years	12		X		X	Yes
MCSminus24	M	37	Anoxia	9 months	9	1	X			Yes
MCSminus25	M	57	TBI	1 month	7	8	X			No

UWS=Unresponsive Wakefulness Syndrome; loca=with auditory localisation; noloca=without auditory localisation; M=male; F=female; CA=cardiac arrest; TBI=traumatic brain injury; SAH=subarachnoid hemorrhage; CRS-R=Coma Recovery Scale Revised; GOSE=Glasgow Outcome Scale Extended (1=Dead; 2=Vegetative State; 3=Less severe disability).

III. Demographic data summary of MCS minus patients and comparison with UWS LOCA and NO-LOCA patients.

	^{18}F-FDG-PET			fMRI			hdEEG		
	MCS minus	p-value (*vs* LOCA)	p-value (*vs* NO-LOCA)	MCS minus	p-value (*vs* LOCA)	p-value (*vs* NO-LOCA)	MCS minus	p-value (*vs* LOCA)	p-value (*vs* NO-LOCA)
**Number of participants**	19			20			11		
**Mean age ± SD**	38±13	p=0.321	**p=0.042***	38±13	p=0.315	**p=0.042***	33±13	p=0.446	p=0.067
**Gender (women/men)**	8/11	p=1.000	p=0.387	7/13	p=0.176	**p=0.010***	4/7	p=1.000	p=1.000
**Etiology (TBI/NTBI)**	11/8	p=0.420	**p=0.013***	12/8	p=0.410	**p=0.002***	7/4	p=0.596	**p=0.014***
**Median time since injury in months (range)**	11 (1-359)	p=0.549	**p=0.045***	10 (1-110)	p=0.218	p=0.064	11 (1-110)	p=0.394	p=0.799

Abbreviations: FDG-PET= fluorodeoxyglucose positron emission tomography; fMRI=functional magnetic resonance imaging; hdEEG=high-density electroencephalography; SD=standard deviation; TBI= traumatic brain injury; NTBI=non-traumatic brain injury. ***statistically significant**

IV. Behavioral acquisitions using the Coma Recovery Scale-Revised.

For each CRS-R evaluation, experienced clinicians assessed all the items of the CRS-R, which ensured to evaluate the auditory localisation every time for each patient. The auditory localisation was assessed as indicated in the CRS-R guidelines: an auditory stimulus was presented for five seconds by the assessor standing behind and out of view of the patient, for a total of four trials, two on each side. Auditory localisation was considered present when an orientation of the head or the eyes towards the stimulus could be observed on both trials for at least one side. Importantly, the CRS-R was systematically performed on the day of the PET, MRI and EEG examinations. The own name was used as it has been shown to elicit more responses than neutral sound.(Cheng *et al.*, 2013)

V. ^{18}F-FDG-PET, MRI and EEG data acquisition parameters, preprocessing and analysis.

FDG-PET: The scan started 30 minutes after an intravenous injection of the tracer (approximately 150-300 MBq of FDG) and lasted 12 minutes. ^{18}FDG-PET images were reconstructed with standard 2x2x2 mm^3 voxels using iterative list mode time-of-flight algorithm and corrections for attenuation, dead-time, random and scatter events were applied. Images for each subject were manually reoriented using Statistical Parametric Mapping (SPM 12, www.fil.ion.ucl.ac.uk/spm). They were then preprocessed including spatial normalization, smoothing (with an isotropic 14 mm FWHM Gaussian filter) and proportional scaling.

Some patients required light sedation during the scanning to prevent excessive movements but this does not affect the results.

MRI: Functional MRI included an Echo Planar Imaging sequence (32 slices, repetition time = 2000 ms, echo time = 30 ms, field of view = 192×192 mm^2, flip angle = 78 degrees, voxel size = 3×3×3 mm^3) and the structural MRI included a T1-weighted 3D gradient echo images (120 slides, repetition time = 2300 ms, echo time = 2.47 ms, voxel size = 1x1x1.2 mm^3, flip angle = 9°, field of view = 256x256 mm^2). Data preprocessing consisted of slice-time correction, realignment, co-registration of functional on structural data, spatial normalization and smoothing with Gaussian isotropic kernel (8mm of full-width-at-half-maximum). For the normalization procedure we used a study-specific template created with DARTEL obtained from patients and HCS.(Ashburner, 2007),(Di Perri *et al.*, 2013),(Peelle *et al.*, 2012) This template was used to minimize normalization difficulty as it decreases the degree of warping necessary for patient brains in the normalization step and reduces the likelihood of misclassification and normalization errors that can occur during the voxel-based morphometry process. For BOLD noise reduction, we used the anatomical component-based noise correction method(Behzadi *et al.*, 2007) as implemented in the *CONN* functional connectivity toolbox(Whitfield-Gabrieli and Nieto-Castanon, 2012). A temporal band-pass filter of 0,008-0,09 Hz was applied on the time series as classically performed in seed-correlation analysis(Greicius *et al.*, 2003; Fox *et al.*, 2005). Regarding

motion correction, we used the artefact detection toolbox (ART; http://nitrc.org/projects/artifact_detect) as described elsewhere(Aubinet *et al.*, 2018), using a composite motion measure. With this approach, a volume was defined as an outlier (artifact) if the largest voxel movement detected was above the specified thresholds. Specifically, an image was defined as an outlier (artifact) image if the head displacement in x, y or z direction was greater than 0.5 mm from the previous frame, or if the rotational displacement was greater than .02 rad from the previous frame, or if the global mean intensity in the image was greater than 3 SD from the mean image intensity for the entire resting scan. Outliers in the global mean signal intensity and motion were subsequently included as nuisance regressors (i.e., one regressor per outlier within the first-level general linear model). In doing so, the temporal structure of the data was not disrupted.

For some patients, a light sedation was required to reduce the severity of movement artefact during the fMRI data acquisition (6/8 LOCA patients and 9/25 NO LOCA patients).

EEG: The recording was acquired while patients were lying in bed, with the eyes open. Data were preprocessed using the following steps: band-pass filtering at 0.05 to 48Hz, baseline correction, independent component analysis to discard the noise components, interpolation of bad channels, average re-referencing and down-sampling to 250Hz. dwPLI is considered as the measure of asymmetry of phase differences between two EEG signals. The participation coefficient measures the between-module connectivity strength as a property of global network integration. Broadly, regions with a high participation coefficient have strong connections to many modules.(Baum *et al.*, 2017)

Electrodes used for each brain region:
Left_frontal:{24, 28, 29, 30, 33, 34, 35, 36, 37, 38, 39, 40, 41, 46, 47, 48, 49, 54, 55};
Right_frontal:{1, 2, 3, 4, 5, 10, 11, 12, 13, 18, 19, 207, 213, 214, 215, 221, 222, 223, 224};
Left_central: {42, 43, 44, 50, 51, 52, 53, 56, 57, 58, 59, 65};
Right_central: {144, 182, 183, 184, 185, 195, 196, 197, 204, 205, 206, 212};
Left_temporal: {62, 63, 64, 68, 69, 70, 71, 74, 75, 83, 84, 94, 95};
Right_temporal: {178, 179, 180, 181, 190, 191, 192, 193, 202, 203, 210, 211, 219};
Left_parietal: {60, 66, 72, 76, 77, 78, 79, 85, 86, 87, 88, 97, 98, 99};
Right_parietal: {141, 142, 143, 152, 153, 154, 155, 161, 162, 163, 164, 171, 172, 173};
Left_occipital: {106, 107, 108, 109, 115, 116};
Right_occipital:{140, 150, 159, 151, 160, 169};
Upper_midline: {6, 7, 8, 9, 14, 15, 16, 17, 20, 21, 22, 23, 25, 26, 27, 32, 186};
Lower_midline: {80, 81, 89, 90, 100, 101, 110, 117, 118, 119, 124, 125, 126, 127, 128, 129, 130, 131, 137, 138, 139, 149}

VI. Seeds coordinates used for fMRI analyses, for the auditory, default mode and fronto-parietal networks.

Networks (reference)	Seeds	MNI coordinates (x, y, z)
Auditory network(Maudoux *et al.*, 2012)	Anterior cingulate cortex	6, -7, 43
	Left precentral gyrus	-53, -6, 8
	Right precentral gyrus	58, -6, 11
	Left superior temporal Gyrus	44, -6, 11
	Right superior temporal Gyrus	44, -6, 11
	Right visual cortex	6, -88, 37
	Left visual cortex	-6, -88, 37
Default mode network(Raichle *et al.*, 2001)	Left cerebellum	-25, -81, -33
	Right cerebellum	25, -81, -33
	Left inferior temporal gyrus	-61, -24, -9
	Right inferior temporal gyrus	58, -24, -9
	Left lateral parietal cortex	-46, -66, 30
	Right lateral parietal cortex	49, -63, 33
	Medial prefrontal cortex	-1, 54, 27
	Posterior cingulate cortex	0, -52, 27
	Thalamus	0, -12, 9
Fronto-parietal network(Fair *et al.*, 2009)	Left dorsolateral prefrontal cortex	-43, 22, 34
	Right dorsolateral prefrontal cortex	43, 22, 34
	Left premotor	-41, 3, 36
	Right premotor	41, 3, 36

	Mild cingulate cortex	0, -29, 30
	Inferior parietal lobule	-51, -51, 36
	Inferior parietal lobule	51, -47, 42
	Left angular gyrus	-31, -59, 42
	Right angular gyrus	30, -61, 39
	Left precuneus	9, -72, 37
	Right precuneus	10, -69, 39

VII. Outcome data of the whole sample of patients (n=125/186)

		Survival	Death	Improvement	Stability	Decline	Missing
**UWS**	LOCA (n=8)	4/7 (57%)	3/7	2/7 (28%)	2/7 (28%)	NA	1/8
	NO-LOCA (n=56)	16/38 (42%)	23/38 (60%)	3/38 (8%)	13/38 (34%)	NA	17/56
**MCS-**	LOCA (n=13)	5/7 (72%)	2/7	0/7 (0%)	4/7 (57%)	1/7 (14%)	6/13
	NO-LOCA (n=15)	8/11 (73%)	3/11	1/11 (9%)	4/11 (36%)	3/11 (27%)	4/15
**MCS+**	LOCA (n=44)	25/30 (84%)	5/30	1/30 (3%)	22/30 (73%)	2/30 (6%)	14/44
	NO-LOCA (n=27)	13/19 (69%)	6/19	0/19 (0%)	10/19 (53%)	3/19 (16%)	8/27
**EMCS**	LOCA (n=18)	10/10 (100%)	0/10	1/10 (10%)	3/10 (30%)	6/10 (60%)	8/18
	NO-LOCA (n=5)	2/3 (67%)	1/3	0/3 (0%)	1/3 (33%)	1/3 (33%)	2/5
**Total (%)**	LOCA (n=83)	44/54 (81.5%)	10/54	4/54 (9%)	36/54 (67%)	4/47 (8.5%)	29/83 (35%)
	NO-LOCA (n=103)	39/71 (56%)	11/71	4/71 (6%)	29/71 (41%)	6/33 (18%)	32/103 (31%)

Abbreviations: NA= not applicable

Survival: GOSE ≠ 1; Improvement: for UWS GOSE > 2, MCS GOSE > 3, EMCS GOSE > 4; Stability: for UWS GOSE = 2, for MCS GOSE = 3, for EMCS GOSE = 4; Decline: for MCS GOSE = 2; EMCS GOSE = 2 or 3.

VIII. ^{18}FDG-PET results. Statistical values of the clusters obtained by the direct comparisons of LOCA and NO-LOCA patients with HCS, as well as LOCA and NO-LOCA with MCS minus patients.

**Contrast**	**Set**		**Cluster**			**Peak**					**MNI coordinates**		
	p	c	*p*(FWE-corr)	equiv k	*p*(unc)	*P*(FWE-corr)	*p*(FDR-corr)	T	equiv Z	*p*(unc)	x,y,z {mm}		
LOCA<HCS	0.263	7	0.000	23190	0.000	0.000	0.000	7.89	6.6	0.000	-2	-38	34
						0.004	0.000	5.28	4.8	0.000	16	-60	16
						0.022	0.001	4.74	4.38	0.000	-10	-58	10
			0.546	855	0.153	0.000	0.000	6.04	5.37	0.000	-6	-14	8
						0.002	0.000	5.49	4.97	0.000	2	-14	6
						0.310	0.006	3.77	3.57	0.000	-22	4	4
			0.955	122	0.599	0.128	0.003	4.13	3.88	0.000	24	14	4
			0.966	92	0.654	0.969	0.034	2.77	2.69	0.004	-26	46	38
			0.991	10	0.908	0.980	0.038	2.71	2.63	0.004	-52	-68	28
			0.994	1	0.979	0.983	0.040	2.69	2.61	0.005	12	2	2
			0.985	30	0.818	0.987	0.042	2.65	2.57	0.005	0	58	-4
NO-LOCA <HCS			0.000	77435	0.000	0.000	0.000	14.27	Inf	0.000	0	-40	34
						0.000	0.000	13.02	Inf	0.000	-6	-68	34
						0.000	0.000	11.47	Inf	0.000	-38	8	56
						0.000	0.000	10.39	Inf	0.000	-26	26	50
						0.000	0.000	9.90	7.72	0.000	-6	-14	8
						0.000	0.000	9.82	7.68	0.000	-46	-58	48

				0.000	0.000	9.80	7.67	0.000	46	10	50
				0.000	0.000	9.79	7.66	0.000	2	-14	6
				0.000	0.000	9.59	7.56	0.000	-42	-60	48
				0.000	0.000	9.50	7.51	0.000	-18	16	62
				0.000	0.000	9.32	7.42	0.000	-8	20	62
				0.000	0.000	9.31	7.41	0.000	-44	12	34
				0.000	0.000	9.25	7.38	0.000	16	4	70
				0.000	0.000	9.20	7.35	0.000	12	10	68
				0.000	0.000	9.04	7.26	0.000	28	26	52
				0.000	0.000	8.89	7.18	0.000	8	20	62
NO-LOCA<MCS-	0.000	23947	0.000	0.000	0.000	6.70	5.55	0.000	-18	-92	20
				0.000	0.000	6.41	5.38	0.000	0	-88	24
				0.002	0.000	5.70	4.92	0.000	4	-86	-4
				0.008	0.000	5.21	4.58	0.000	8	-64	8
				0.123	0.003	4.18	3.82	0.000	24	-58	60
				0.331	0.007	3.72	3.45	0.000	42	-28	64
				0.334	0.007	3.71	3.45	0.000	38	-28	66
				0.443	0.009	3.55	3.32	0.000	18	-16	68
				0.521	0.011	3.45	3.23	0.001	-10	-30	72
				0.925	0.036	2.82	2.69	0.004	42	-70	2
	0.325	1685	0.111	0.238	0.005	3.88	3.59	0.000	56	-6	8
				0.301	0.006	3.77	3.49	0.000	52	-22	10
				0.309	0.006	3.75	3.48	0.000	32	-2	14

IX. fMRI results. Comparaison between LOCA and HCS (on the left) and between NO-LOCA and HCS (on the right) of the correlation between the auditory (first row), default mode (second row) and fronto-parietal (third row) networks and the time series from all other brain voxels. The blue spots show significantly decreased functional connectivity between patients and HCS. Statistical maps are thresholded at $p<0.05$ false discovery rate corrected at non-parametric cluster-mass with clusters made of voxels surviving a $p<0.001$ (whole-brain level).

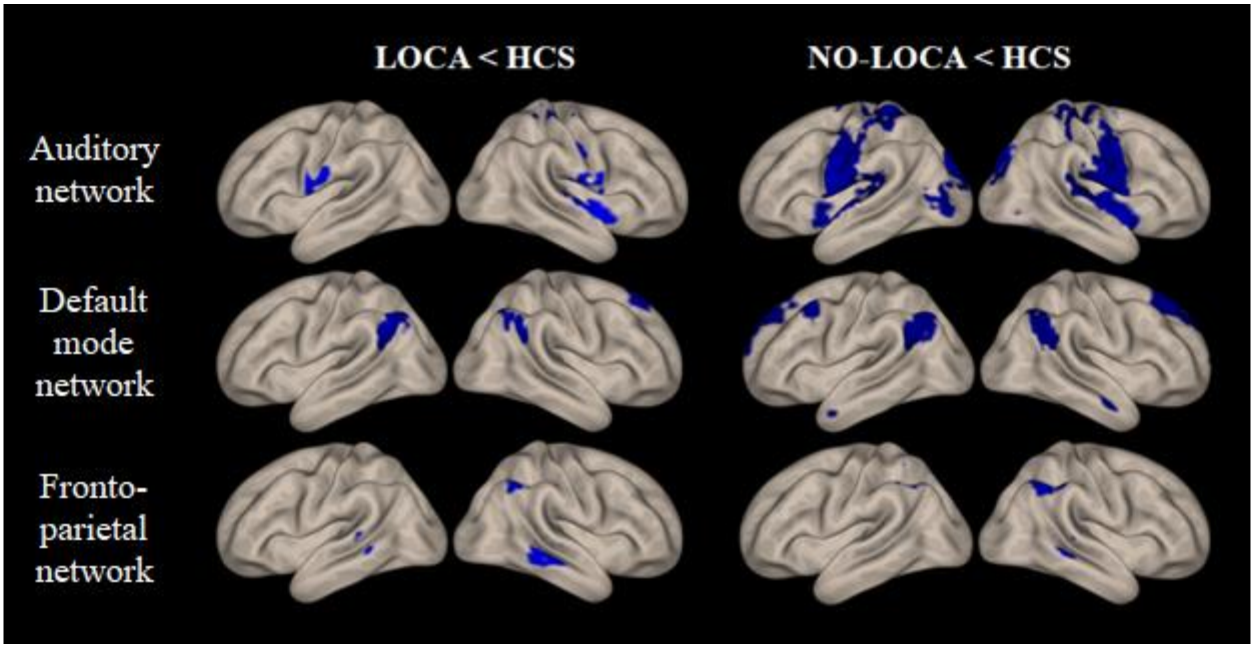

X. fMRI results: Statistical values of the clusters obtained by the comparisons of LOCA patients and HCS, and NO-LOCA patients and HCS for the auditory, default mode and fronto-parietal networks; and the comparison of LOCA and NO-LOCA, for the fronto-parietal network only.

Network	Contrast	Cluster size	t-value	MNI coordinates			Regions
				x	y	z	

**Auditory**	**LOCA < HCS**	1088	5,078	64	2	0	Superior Temporal Gyrus, anterior division
			4,843	66	4	20	Precentral Gyrus
			4,799	42	-2	-6	Insular Cortex
		412	4,972	-62	-4	18	Precentral Gyrus
			3,559	-62	-26	10	Planum Temporale
		167	4,951	0	-4	60	Juxtapositional Lobule Cortex (formerly Supplementary Motor Cortex)
		110	4,685	42	-10	34	Precentral Gyrus
		118	4,409	10	-86	28	Cuneal Cortex
		156	4,274	12	-64	-12	Lingual Gyrus
		234	4,195	30	-34	60	Postcentral Gyrus
			3,685	40	-18	70	Precentral Gyrus
	**NO-LOCA < HCS**	6025	7,794	16	-82	34	Lateral Occipital Cortex, superior division
			6,397	4	-86	18	Cuneal Cortex
			5,933	16	-56	-12	Lingual Gyrus
		4902	7,083	42	-8	-8	Insular Cortex
			6,789	60	-2	0	Planum Polare
			6,338	42	-14	34	Postcentral Gyrus
		5085	6,581	-60	-4	4	Planum Polare
			6,258	-50	-8	28	Precentral Gyrus
			6,150	-54	-28	10	Planum Temporale
		639	5,490	-18	-64	-14	Lingual Gyrus
		2409	5,441	30	-34	58	Postcentral Gyrus
			5,395	4	-8	60	Juxtapositional Lobule Cortex (formerly Supplementary Motor Cortex)
			4,195	-6	-14	42	Cingulate Gyrus, anterior division
		514	4,828	-42	-86	-6	Lateral Occipital Cortex, inferior division
			4,644	-42	-84	14	Lateral Occipital Cortex, superior division
**Default mode**	**LOCA < HCS**	1125	6,597	4	-58	30	Precuneus Cortex
		822	5,304	-48	-64	30	Lateral Occipital Cortex, superior division
			3,831	-42	-70	50	Lateral Occipital Cortex, superior division
		430	4,847	0	50	24	Paracingulate Gyrus
			4,143	0	46	4	Cingulate Gyrus, anterior division
		451	4,748	58	-56	28	Angular Gyrus
			3,901	42	-64	46	Lateral Occipital Cortex, superior division
	**NO-LOCA < HCS**	2076	9,312	4	-52	28	Cingulate Gyrus, posterior division
			3,842	-4	-68	18	Precuneus Cortex
		1314	6,818	-48	-64	30	Lateral Occipital Cortex, superior division
			5,386	-38	-74	46	Lateral Occipital Cortex, superior division
			3,347	-64	-62	18	Angular Gyrus
		3220	6,727	0	50	4	Paracingulate Gyrus
			5,937	0	50	26	Paracingulate Gyrus
			5,377	18	32	48	Superior Frontal Gyrus
		963	6,414	58	-56	28	Angular Gyrus
			4,445	42	-64	48	Lateral Occipital Cortex, superior division
		369	5,768	-38	16	52	Middle Frontal Gyrus
		206	4,877	-56	4	-32	Temporal Pole
			4,370	-44	16	-44	Temporal Pole
		249	4,835	66	-2	-24	Middle Temporal Gyrus, anterior division
			3,796	56	4	-42	Temporal Pole
		90	4,481	-18	-26	-12	Parahippocampal Gyrus, posterior division
**Fronto-parietal**	**LOCA < HCS**	407	4,891	66	-34	-6	Middle Temporal Gyrus, posterior division
		121	4,760	-14	-88	-24	Occipital Fusiform Gyrus
		120	4,497	-66	-44	-2	Middle Temporal Gyrus, temporooccipital part
		113	4,387	28	14	66	Superior Frontal Gyrus
		261	4,285	52	-46	52	Supramarginal Gyrus, posterior division
			3,450	50	-58	36	Angular Gyrus
	**NO-LOCA < HCS**	182	5,650	66	-38	-2	Middle Temporal Gyrus, temporooccipital part
		884	5,358	48	-50	54	Angular Gyrus
			3,696	64	-48	42	Angular Gyrus
		164	4,990	6	-10	10	Right Thalamus
		477	4,829	-50	-52	54	Supramarginal Gyrus, posterior division
			3,760	-32	-64	54	Lateral Occipital Cortex, superior division
	**LOCA>NO-LOCA**	323	5,055	-34	-84	26	Occipital pole
			4,155	-18	-94	18	Lateral occipital cortex

XI. List of all pharmacological agents acting on the nervous system, taken by the patients who underwent neuroimaging/electrophysiological examinations

UWS-loca1	Depakine, Rivotril
UWS-loca2	Amantadine
UWS-loca3	/
UWS-loca4	Lormetazepam, Phenobarbital, Lamictal, Keppra, Rivotril, Diazepam
UWS-loca5	Keppra, Lamictal, Lorazepam
UWS-loca6	Depakine, Dafalgan, Keppra, Gardenal
UWS-loca7	Mantadix, Rivotril
UWS-loca8	Amantadine, Citalopram, Gabapentine
UWS-noloca1	Keppra
UWS-noloca2	/
UWS-noloca3	Hydromorphone, Fentanyl, Amantadine, Levetiracetam, Clonidine, Escitalopram
UWS-noloca4	Keppra
UWS-noloca5	Depakin
UWS-noloca6	Morphine
UWS-noloca7	/
UWS-noloca8	/
UWS-noloca9	Keppra, Amantadine, Trazodone, Depakine
UWS-noloca10	Keppra
UWS-noloca11	Depakine, Levetiracetam, Tegretol, Amantadine
UWS-noloca12	/
UWS-noloca13	/
UWS-noloca14	Lexomil, Lyrica, Contramal
UWS-noloca15	/
UWS-noloca16	Epanutin, Keppra
UWS-noloca17	Seroxat, Rifotril, Tegretol
UWS-noloca18	Keppra, Tegretol
UWS-noloca19	Depakine
UWS-noloca20	Trazolan, Durogesic
UWS-noloca21	Amantadine, Valium
UWS-noloca22	Lysanxia
UWS-noloca23	Valium
UWS-noloca24	Keppra, Amantadine, Dominal
UWS-noloca25	Diphantoine, Lioresal, Diazepam, Lysanxia, Depakine
UWS-noloca26	Keppra, Amantadine
UWS-noloca27	Amantadine
UWS-noloca28	Keppra, Lysanxia, Zolpidem, Trazolan
UWS-noloca29	Keppra, Rivotril, Cerebrolysin, Piracetam
UWS-noloca30	Gardenal, Keppra, Amantadine, Diphantoine
UWS-noloca31	Prolopa
UWS-noloca32	/
UWS-noloca33	Clonazepam
UWS-noloca34	Keppra
UWS-noloca35	Neurontin, Depakine
MCSminus1	Oxynorm instant, Sipralexa, Durogesic
MCSminus2	Amantadine
MCSminus3	Keppra
MCSminus4	/
MCSminus5	Xanax
MCSminus6	Oxazepam, Depakine, Valium
MCSminus7	Valium, Serlain
MCSminus8	/
MCSminus9	Dominal forte, Rivotril
MCSminus10	Keppra
MCSminus11	Xanax, Midazolam
MCSminus12	Keppra, Valproate
MCSminus13	Transtec, Trazolan, Loramet, Lysanxia
MCSminus14	Keppra, Diphantoine
MCSminus15	Depakine
MCSminus16	/
MCSminus17	Keppra, Sipralexa
MCSminus18	Depakine, Keppra
MCSminus19	Prazepam, Keppra, Amantadine
MCSminus20	Amantadine, Seroxat, Theralene
MCSminus21	/
MCSminus22	Keppra, Sipralexa, Trazolan, Amantadine, Lysanxia
MCSminus23	Rivotril, Diphantoine, Keppra
MCSminus24	Epanutin

References

Ashburner J. A fast diffeomorphic image registration algorithm. Neuroimage 2007; 38: 95–113.

Aubinet C, Larroque SK, Heine L, Martial C, Majerus S, Laureys S, et al. Clinical subcategorization of minimally conscious state according to resting functional connectivity. Hum Brain Mapp 2018; 39: 4519–4532.

Baum GL, Ciric R, Roalf DR, Betzel RF, Moore TM, Shinohara RT, et al. Modular Segregation of Structural Brain Networks Supports the Development of Executive Function in Youth. Curr Biol 2017; 27: 1561-1572.e8.

Behzadi Y, Restom K, Liau J, Liu TT. A component based noise correction method (CompCor) for BOLD and perfusion based fMRI. Neuroimage 2007; 37: 90–101.

Cheng L, Gosseries O, Ying L, Hu X, Yu D, Gao H, et al. Assessment of localisation to auditory stimulation in post-comatose states: Use the patient's own name. BMC Neurol 2013; 13

Fair DA, Cohen AL, Power JD, Dosenbach NUF, Church JA, Miezin FM, et al. Functional brain networks develop from a 'local to distributed' organization. PLoS Comput Biol 2009; 5: 14–23.

Fox MD, Snyder AZ, Vincent JL, Corbetta M, Van Essen DC, Raichle ME. The human brain is intrinsically organized into dynamic, anticorrelated functional networks. Proc Natl Acad Sci U S A 2005; 102: 9673–8.

Greicius MD, Krasnow B, Reiss AL, Menon V. Functional connectivity in the resting brain: a network analysis of the default mode hypothesis. Proc Natl Acad Sci U S A 2003; 100: 253–8.

Maudoux A, Lefebvre P, Cabay JE, Demertzi A, Vanhaudenhuyse A, Laureys S, et al. Auditory resting-state network connectivity in tinnitus: A functional MRI study. PLoS One 2012; 7: 1–9.

Peelle JE, Cusack R, Henson RNA. Adjusting for global effects in voxel-based morphometry: Gray matter decline in normal aging. Neuroimage 2012; 60: 1503–1516.

Di Perri C, Bastianello S, Bartsch AJ, Pistarini C, Maggioni G, Magrassi L, et al. Limbic hyperconnectivity in the vegetative state. Neurology 2013; 81: 1417–1424.

Raichle ME, MacLeod AM, Snyder AZ, Powers WJ, Gusnard DA, Shulman GL. A default mode of brain function. Proc Natl Acad Sci U S A 2001; 98: 676–82.

Whitfield-Gabrieli S, Nieto-Castanon A. *Conn* : A Functional Connectivity Toolbox for Correlated and Anticorrelated Brain Networks. Brain Connect 2012; 2: 125–141.
